# Fentanyl or morphine for persistent dyspnoea in COPD: a multicentre randomised crossover trial

**DOI:** 10.1183/13993003.01431-2025

**Published:** 2026-08-06

**Authors:** Marlies van Dijk, Kris J.M. Mooren, Wendy van Beurden-Moeskops, Roxane Heller-Baan, Sander M. de Hosson, Remge M. Pieterman, Wai Yee Lam-Wong, Liesbeth Peters, Karin Pool, Jan-Willem K. van den Berg, Huib A.M. Kerstjens

**Affiliations:** 1 Department of Pulmonary Diseases, University of Groningen, University Medical Center Groningen, Groningen, The Netherlands; 2 Groningen Research Institute of Asthma and COPD (GRIAC), University of Groningen, Groningen, The Netherlands; 3 Department of Pulmonary Diseases, Spaarne Gasthuis, Haarlem, The Netherlands; 4 Department of Pulmonary Diseases, Medisch Spectrum Twente, Enschede, The Netherlands; 5 Department of Pulmonary Diseases, Ikazia Ziekenhuis, Rotterdam, The Netherlands; 6 Department of Pulmonary Diseases, Wilhelmina Ziekenhuis Assen, Assen, The Netherlands; 7 Department of Pulmonary Diseases, Ommelander Ziekenhuis Groningen, Scheemda, The Netherlands; 8 Department of Pulmonary Diseases, Elkerliek Ziekenhuis, Helmond, The Netherlands; 9 Department of Pulmonary Diseases, Noordwest Ziekenhuisgroep, Alkmaar, The Netherlands; 10 Department of Pulmonary Diseases, Rode Kruis Ziekenhuis, Beverwijk, The Netherlands; 11 Department of Pulmonary Diseases, Isala Klinieken, Zwolle, The Netherlands

## Abstract

**Background:**

One of the most prevalent and impactful symptoms in COPD is persistent dyspnoea, occurring in the majority of patients with advanced COPD. Opioids, mostly morphine, have been investigated and suggested as treatment in palliative care guidelines. However, in recent years multiple trials on morphine for persistent dyspnoea have shown no clinical benefit, and there are concerns regarding adverse events. Transdermal fentanyl has not yet been investigated for this indication.

**Methods:**

We performed a multicentre, crossover, double-dummy randomised controlled trial, investigating transdermal fentanyl (12 μg·h^−1^), morphine (10 mg *per os* twice daily) and placebo, during three treatment periods of 2 weeks (including a 3-day washout period). Patients with persistent dyspnoea despite optimal therapy and modified Medical Research Council dyspnoea scale score ≥3 caused by COPD, forced expiratory volume in 1 s (FEV_1_) <50% predicted and stable disease were included. The primary end-point was change in mean daily dyspnoea score on a Numeric Rating Scale (NRS). Secondary outcomes were change in worst daily dyspnoea (NRS), health status (Clinical COPD Questionnaire), anxiety (Hospital Anxiety and Depression Scale-Anxiety), hypercapnia (capillary blood gas), mastery (Chronic Respiratory Questionnaire-Mastery) and sleep quality (NRS).

**Results:**

Between April 2019 and November 2024, 59 randomisations were performed in 58 participants (mean±sd 69±7 years; 54% female; FEV_1_ 33±10% predicted); 37 participants completed the study. The main reasons for study discontinuation were exacerbations (n=13) and side-effects (n=7). There were no significant differences between both fentanyl and morphine compared to placebo for mean and worst dyspnoea, sleep, health status, mastery, anxiety, hypercapnia or side-effects (except for a possible difference in vomiting).

**Conclusion:**

This study demonstrated no positive effect of transdermal fentanyl nor oral morphine compared to placebo in the treatment of persistent dyspnoea in COPD.

## Shareable abstract

**This trial investigated low-dose transdermal fentanyl and oral morphine compared to placebo for persistent dyspnoea in stable COPD. No significant differences were found for dyspnoea, sleep, health status, anxiety, hypercapnia or side-effects.** https://bit.ly/4m4TjeS

## Graphical abstract

**Figure:**
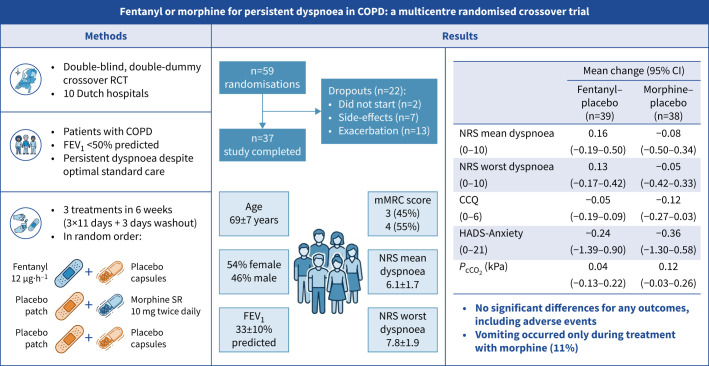
Overview of study design and main study outcomes. RCT: randomised controlled trial; FEV_1_: forced expiratory volume in 1 s; SR: sustained release; mMRC: modified Medical Research Council dyspnoea scale; NRS: Numeric Rating Scale; CCQ: Clinical COPD Questionnaire; HADS: Hospital Anxiety and Depression Scale; *P*_cCO_2__: capillary carbon dioxide tension.

## Introduction

Dyspnoea is one of the most prevalent and burdensome symptoms in patients with advanced COPD, affecting up to 94% of individuals in the final years of life [1, 2]. Dyspnoea is considered persistent when the symptom persists despite optimal standard care [1]. It significantly impairs health-related quality of life, limits physical activity, and is associated with anxiety and depression. Furthermore, persistent dyspnoea not only impacts the life of patients, but also of their loved ones [2]. Since the number of patients with COPD worldwide is still increasing, there will be an increasing number of patients suffering from persistent dyspnoea caused by COPD. This underscores an urgent clinical need for effective and accessible therapeutic options to alleviate the distress associated with persistent dyspnoea.

Advanced therapeutic options, including pulmonary rehabilitation, non-invasive ventilation, lung volume reduction treatments and lung transplantation, can improve dyspnoea and quality of life in patients with severe COPD [3]. However, due to strict eligibility criteria, high healthcare costs, varying availability and in some cases scarcity, these treatments are not available for a large proportion of patients with advanced COPD. This underlines the need for a widely available, preferably low-cost, treatment for persistent dyspnoea in COPD.

Low-dose opioids have been investigated for persistent dyspnoea, and some studies have demonstrated a positive effect of opioids on dyspnoea [4–6]. This treatment option has therefore made its way into several guidelines, among which the Dutch guideline for palliative care in COPD [7]. Nevertheless, concerns persist regarding the clinical significance of the observed effects, as well as the potential for adverse events associated with opioid use.

To date, the majority of clinical research on opioid therapy for persistent dyspnoea in COPD has focused on oral morphine. To the best of our knowledge, no randomised controlled trials (RCTs) have been conducted to investigate the efficacy of long-acting transdermal fentanyl in this context. This is relevant since there are indications that fentanyl is associated with fewer gastrointestinal side-effects compared to morphine, and patients may prefer a patch to oral medication [8]. Therefore, we aimed to investigate the effects of transdermal fentanyl on persistent dyspnoea in COPD. For this purpose we designed a double-dummy crossover RCT investigating transdermal fentanyl, sustained-release morphine and placebo. This might appear unconventional, since it is common to only make a comparison of the investigative drug to the standard of treatment. However, while designing our study in 2017, we considered the evidence on morphine for persistent dyspnoea to be of low quality [9], and therefore believed it important to include a placebo in the study.

Our study hypothesis was that both transdermal fentanyl and oral morphine are more effective for persistent dyspnoea in COPD than placebo, and that fentanyl has less side-effects than morphine. In addition, we aimed to investigate the effect of both fentanyl and morphine compared to placebo on health status, anxiety, sleep quality, mastery, hypercapnia and adverse events.

## Materials and methods

### Study design and oversight

This was a multicentre double-blind, double-dummy crossover three-arm RCT in 10 hospitals in the Netherlands. The study was performed in accordance with the Declaration of Helsinki, and was approved by the University Medical Center Groningen (Groningen, the Netherlands) Ethics Committee (METc 2018/567). Written informed consent was obtained from all participants. The study protocol including the statistical analysis plan was published previously [9] and the study is registered at ClinicalTrials.gov (NCT03834363). The study design was discussed in the Patient Advisory Board of the Groningen Research Institute for Asthma and COPD on 6 September 2018.

### Participants

Patients with stable COPD Global Initiative for Chronic Obstructive Lung Disease (GOLD) class III or IV and a modified Medical Research Council (mMRC) dyspnoea scale score ≥3 who perceived dyspnoea caused by COPD despite optimal standard therapy were eligible. Participants who had a moderate (requiring oral corticosteroids and/or antibiotics) or severe (requiring hospital admission) exacerbation during the trial were discontinued from the trial, since we aimed to investigate the effect of the study treatment in stable COPD, and an exacerbation would influence the daily outcome measurements. After patients were stable during 8 weeks they were allowed to participate in the study one more time [9]. A complete overview of inclusion and exclusion criteria is presented in supplementary table S1. All usual pharmacological and non-pharmacological care was continued during the trial. Whether the patients were receiving optimal standard therapy, and therefore had what we labelled “persistent dyspnoea”*,* was assessed by their respiratory physician. Participants were not allowed to participate in pulmonary rehabilitation during the trial, since this could also improve their dyspnoea.

### Randomisation and intervention

**FIGURE 1:**
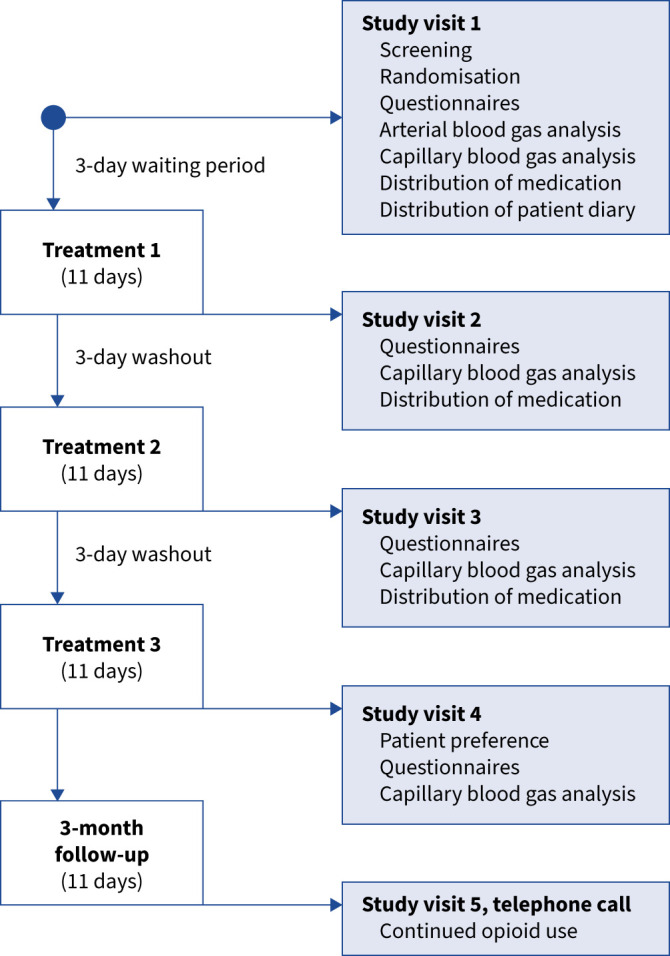
Overview of study visits and treatment periods.

Randomisation was tailormade for this study using a web-based platform (ALEA DM version 17.1; www.aleaclinical.eu). In this three-way crossover trial, participants received all three treatments, in random order. At the randomisation visit participants were allocated to one of the six possible treatment sequences, stratified for study location. The random allocation sequence and assignment was generated by a data manager from the Department of Medical and Information Technology within the University Medical Center Groningen using ALEA. Participants were enrolled by the site principal investigators and research nurses, who also distributed the allocated double-blind medication. All participants and all healthcare providers (including local pharmacy personnel) and members of the research teams were blinded to the study treatment. There was an emergency unblinding procedure in place, which could be effectuated by a local pharmacy staff member. Participants were treated during three periods of 11 days, with a 3-day washout period in between. Study treatment periods consisted of either morphine sustained-release capsules 10 mg twice daily combined with a placebo patch (applied every 72 h), placebo capsules twice daily combined with a 12 μg·h^−1^ fentanyl patch (applied every 72 h) or placebo capsules twice daily combined with a placebo patch (applied every 72 h). To manage common opioid-related side-effects, patients were co-prescribed metoclopramide 10 mg as needed (up to three times daily) for nausea and macrogol/electrolyte solution (13.7 g daily, adjusted as needed) for constipation. To optimise protocol adherence, participants received a reminder phone call 3 days after each study visit, instructing them to initiate the next treatment period the following morning. This ensured an 11-day treatment window before the subsequent visit, with the required 3-day washout period in between. An overview of study visits and treatment periods is provided in figure 1, and has been previously described in detail in the published study protocol [9]. During study participation all regular medical care and treatments were continued.

### Baseline and outcome measures

The primary end-point was change in mean daily dyspnoea sensation during each study period. Secondary end-points included change during each study period in worst daily dyspnoea sensation, health status, anxiety symptoms, mastery, capillary blood gas parameters (pH, carbon dioxide and bicarbonate) and sleep quality. Additionally, the occurrence of side-effects, patient preference and continued opioid use were assessed 3 months after completion of the study intervention.

Change in mean daily dyspnoea, worst daily dyspnoea sensation and sleep quality were assessed once daily on a Numeric Rating Scale (NRS) in a patient diary (supplementary figures S1 and S2) [10]. The questions for dyspnoea in the patient diary (translated from Dutch to English) were: “Please indicate with a number how much, on average, you have suffered from dyspnoea in the past 24 hours” and “Please indicate with a number how severe the worst level of dyspnoea you have felt in the past 24 hours was”. Health status was evaluated daily in a patient diary with the Chronic COPD Questionnaire (CCQ) and mastery was evaluated during every study visit with the Chronic Respiratory Questionnaire-Mastery domain (CRQ-M) [11, 12]. Anxiety was assessed during every study visit with the Hospital Anxiety and Depression Scale-Anxiety subscale (HADS-A) [13]. Higher scores on the NRS, CCQ and HADS-A indicate a greater symptom burden. Side-effects were reported daily in a patient diary and reviewed systematically during each study visit. Specifically named and systematically asked side-effects were obstipation, nausea, vomiting, drowsiness and dizziness. Treatment preference was assessed at the conclusion of the final treatment period, while continued opioid use was evaluated *via* telephone follow-up 3 months post-treatment. Furthermore, spirometry was performed to confirm the diagnosis of COPD GOLD III or IV once before inclusion in accordance with European Respiratory Society/American Thoracic Society guidelines [14], and venous blood was drawn once before inclusion to determine the estimated glomerular filtration rate because of possible opioid accumulation with renal failure [9].

### Statistics

The power calculation was based on the difference between opioid and placebo. The minimal clinically important difference for the NRS score is 1 point, with a standard deviation of 2.0 points [15]. With a two-sided α of 5% and a power of 90% in a crossover design, a sample size of 44 would be needed. Because the study population consists of patients with advanced COPD, whom we assessed to be fragile, we aimed to recruit 60 participants allowing for dropouts.

The primary analysis was performed on an intention-to-treat basis. For the NRS scores of mean dyspnoea, worst dyspnoea and sleep, means of measurements of days 7–14 per treatment period were used for the analysis in order to prevent influence of any carryover medication effects (in addition to the washout period). For the statistical analysis, the outcomes for both fentanyl and morphine were compared to placebo. The paired t-test was used for normally distributed data, otherwise Wilcoxon's signed-rank test was used. Side-effects were tested by comparison of proportions by the McNemar test between all three arms. Data are presented as mean with standard deviation, unless otherwise specified [9].

## Results

**FIGURE 2:**
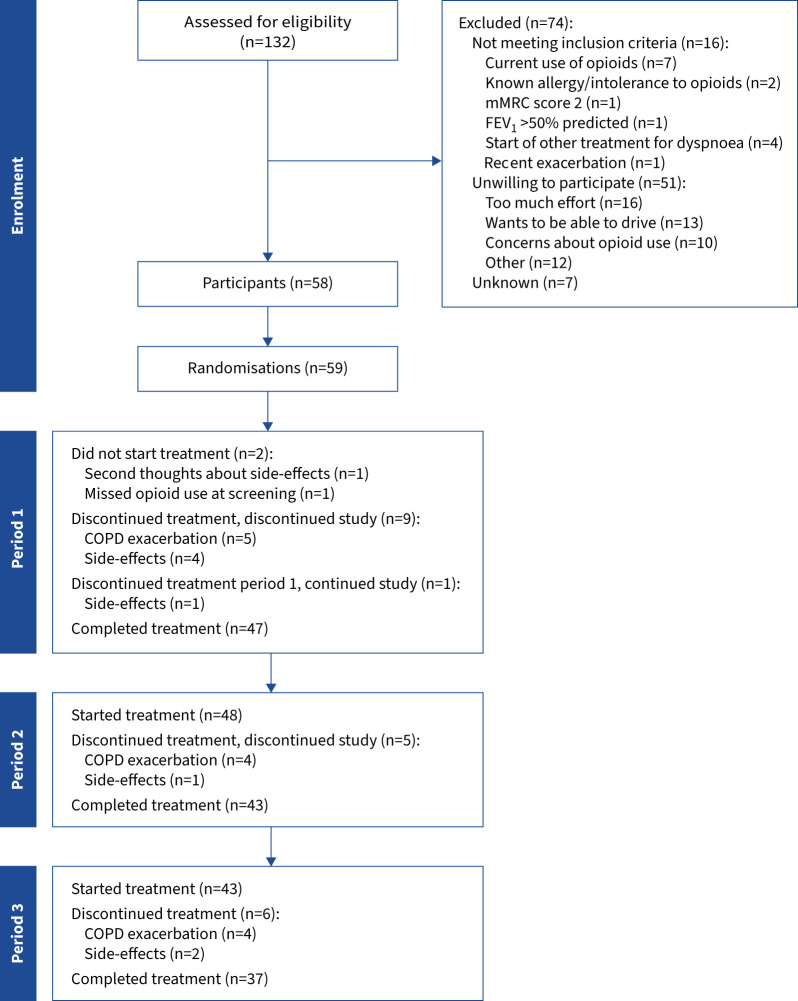
Study flowchart. mMRC: modified Medical Research Council dyspnoea scale; FEV_1_: forced expiratory volume in 1 s.

Between November 2019 and April 2024, 132 patients with COPD were assessed for study eligibility, of which 58 patients were randomised in the study (figure 2). One participant was randomised twice, but dropped out both times because of an exacerbation. 37 participants completed the study. The main reasons for study discontinuation were exacerbations (n=13; n=5 during fentanyl, n=5 during morphine and n=3 during placebo) and side-effects (n=7; n=3 during fentanyl, n=1 during morphine and n=3 during placebo). One patient discontinued study medication during the first treatment period, but did continue treatment in the second and third treatment period. Detailed information on study discontinuation is presented in figure 2. One serious adverse event (SAE), a COPD exacerbation, occurred during treatment with morphine, and was defined as a SAE due to hospital admission. This SAE was resolved completely without the need for (non-)invasive ventilation. The other 12 exacerbations were moderate exacerbations. None of the exacerbations were deemed to be treatment related. No participants died during study participation.

The mean age of the participants was 69±7 years, 54% were female, mean FEV_1_ was 33±10% predicted and median mMRC score was 4 points. All baseline characteristics are presented in table 1 and supplementary table S2.

**TABLE 1:** Baseline characteristics of participants

**Age (years)**	69±7
**Sex**	
Female	32 (54)
Male	26 (44)
**BMI (kg·m^−2^)**	25±5
**Smoking status**	
Former smoker	50 (86)
Current smoker	8 (14)
**Smoking history (pack-years)**	49±36
**Comorbidities (n)**	1 (0–5)
**COPD exacerbations (past year)**	
0	19 (33)
1	13 (22)
2	16 (28)
≥3	10 (17)
**Current medication use**	
LABA	48 (83)
LAMA	47 (81)
ICS	50 (86)
As-needed SABA	34 (59)
As-needed SAMA	42 (67)
OCS	9 (16)
Azithromycin	14 (24)
Antidepressants	8 (14)
Anxiolytic	3 (5.2)
Sleep medication	2 (3.4)
**Long-term oxygen therapy**	22 (37)
**Pulmonary function**	
FEV_1_ (L)	0.88±0.33
FEV_1_ (% pred)	33±10
FEV_1_/FVC	0.36±0.13
**Blood gas analysis**	
*P*_aO_2__ (kPa)	9.6±1.7
*P*_aCO_2__ (kPa)	5.8±0.9
*P*_cCO_2__ (kPa)	5.9±1.0
**mMRC dyspnoea scale score**	
3	26 (45)
4	32 (55)
**Questionnaires (past 7 days)**	
NRS mean dyspnoea (0–10)	6.1±1.7
NRS worst dyspnoea (0–10)	7.8±1.9
NRS sleep (0–10)	4.1±3.1
CCQ (0–6)	2.8±0.7
CRQ-M (0–7)	4.8±1.2
HADS-A (0–21)	5.3±2.7

Data are presented as mean±sd, n (%) or median (minimum–maximum). BMI: body mass index; LABA: long-acting β-agonist; LAMA: long-acting muscarinic antagonist; ICS: inhaled corticosteroid; SABA: short-acting β-agonist; SAMA: short-acting muscarinic antagonist, OCS: oral corticosteroid; FEV_1_: forced expiratory volume in 1 s; FVC: forced vital capacity; *P*_aO_2__: arterial oxygen tension; *P*_aCO_2__: arterial carbon dioxide tension; *P*_cCO_2__: capillary carbon dioxide tension; mMRC: modified Medical Research Council; NRS: Numeric Rating Scale; CCQ: Clinical COPD Questionnaire; CRQ-M: Chronic Respiratory Questionnaire-Mastery; HADS-A: Hospital Anxiety and Depression Scale-Anxiety.

39 participants completed both the fentanyl and placebo treatment period; 38 participants completed both the morphine and placebo treatment period. There were no statistically significant differences between fentanyl compared to placebo nor morphine compared to placebo with respect to mean daily dyspnoea score (primary end-point) nor worst dyspnoea score, sleep quality, health status, mastery, anxiety or capillary blood gas parameters (tables 2 and 3).

**TABLE 2:** Outcomes for fentanyl compared to placebo

	Fentanyl (n=39)	Placebo (n=39)	Mean difference	p-value
**NRS mean dyspnoea (0–10) (n=39)**	5.3 (4.8–5.9)	5.2 (4.7–5.7)	0.16 (−0.19–0.50)	0.36
**NRS worst dyspnoea (0–10) (n=39)**	6.0 (5.3–6.7)	5.9 (5.2–6.6)	0.13 (−0.17–0.42)	0.39
**NRS sleep (0–10) (n=39)**	3.6 (2.9–4.3)	3.5 (2.9–4.1)	0.17 (−0.36–0.69)	0.52
**CCQ (0–6) (n=39)**	2.6 (2.3–2.8)	2.6 (2.4–2.8)	−0.05 (−0.19–0.09)	0.46
**CRQ-M (0–7) (n=38)**	4.8 (4.3–5.2)	5.0 (4.6–5.5)	−0.25 (−0.63–0.13)	0.20
**HADS-A (0–21) (n=37)**	5.2 (4.3–6.2)	5.5 (4.5–6.4)	−0.24 (−1.39–0.90)	0.67
***P*_cCO_2__** **(kPa) (n=34)**	5.6 (5.2–6.0)	5.6 (5.3–5.9)	0.04 (−0.13–0.22)	0.61

Data are presented as mean (95% confidence intervals), unless otherwise stated. NRS: Numeric Rating Scale; CCQ: Clinical COPD Questionnaire; CRQ-M: Chronic Respiratory Questionnaire-Mastery; HADS-A: Hospital Anxiety and Depression Scale-Anxiety; *P*_cCO_2__: capillary carbon dioxide tension. The comparison between fentanyl and placebo was tested with the paired t-test. p<0.05 was considered statistically significant.

**TABLE 3:** Outcomes for morphine compared to placebo

	Morphine (n=38)	Placebo (n=38)	Mean difference	p-value
**NRS mean dyspnoea (0–10) (n=38)**	5.1 (4.6–5.7)	5.2 (4.7–5.8)	−0.08 (−0.50–0.34)	0.71
**NRS worst dyspnoea (0–10) (n=38)**	5.9 (5.2–6.7)	6.0 (5.3–6.7)	−0.05 (−0.42–0.33)	0.80
**NRS sleep (0–10) (n=38)**	3.2 (2.5–3.8)	3.2 (2.6–3.8)	−0.02 (−0.39–0.36)	0.92
**CCQ (0–6) (n=38)**	2.5 (2.5–2.8)	2.7 (2.4–2.9)	−0.12 (−0.27–0.03)	0.10
**CRQ-M (0–7) (n=37)**	4.8 (4.3–5.2)	5.0 (4.5–5.5)	−0.23 (−0.52–0.06)	0.12
**HADS-A (0–21) (n=36)**	5.0 (4.2–5.8)	5.4 (4.4–6.3)	−0.36 (−1.30–0.58)	0.44
***P*_cCO_2__** **(kPa) (n=33)**	5.7 (5.3–6.1)	5.6 (5.2–5.9)	0.12 (−0.03–0.26)	0.12

Data are presented as mean (95% confidence intervals), unless otherwise stated. NRS: Numeric Rating Scale; CCQ: Clinical COPD Questionnaire; CRQ-M: Chronic Respiratory Questionnaire-Mastery; HADS-A: Hospital Anxiety and Depression Scale-Anxiety; *P*_cCO_2__: capillary carbon dioxide tension. The comparison between morphine and placebo was tested with the paired t-test. A p<0.05 was considered statistically significant.

At each visit, we asked patients systematically for the major known side-effects of opioids. Vomiting occurred more often during the treatment period with morphine, *i.e.* in five participants (11%) *versus* zero participants during the fentanyl and placebo period. However, because vomiting did not occur during the fentanyl and placebo period it could not be tested whether this difference was statistically different. There were no significant differences with respect to obstipation, nausea, drowsiness and dizziness between groups (table 4). A comprehensive overview of all adverse events, as recorded in patient diaries, is provided in supplementary table S3; there were no marked imbalances.

**TABLE 4:** Adverse events for each treatment, specifically asked during each study visit

	Fentanyl (n=48)	Morphine (n=45)	Placebo (n=44)
**Obstipation**	9 (19)	12 (27)	9 (21)
**Nausea**	12 (25)	10 (22)	9 (19)
**Vomiting**	0 (0)	5 (11)^#^	0 (0)
**Drowsiness**	9 (19)	8 (18)	6 (14)
**Dizziness**	6 (13)	4 (9)	8 (18)

Data are presented as n (%). ^#^: because vomiting did not occur during the fentanyl and placebo period it could not be tested whether this difference was statistically different. The comparison between all treatments was tested with the McNemar test. There were no significant differences between groups.

The preferred treatment period was fentanyl in 13 participants (38%), morphine in 13 participants (38%) and placebo in eight participants (24%). 12 participants (33%) were using opioids 3 months after study treatment completion (fentanyl in five participants, sustained-release morphine in six participants and short-acting morphine in one participant).

As a *post hoc* analysis we analysed how many responders there were for each treatment arm with respect to the primary end-point, *i.e.* the NRS mean dyspnoea score. A responder was defined as a participant with a reduction of ≥1.0 points on the NRS compared to the baseline score [15]. For the NRS mean dyspnoea, the responder rate was 13/40 (33%) for fentanyl, 16/38 (42%) for morphine and 18/39 (46%) for placebo.

## Discussion

To the best of our knowledge, this is the first RCT investigating transdermal fentanyl for persistent dyspnoea. At the time when we were designing our study, morphine was part of guidelines as a treatment option for persistent dyspnoea. However, the effect of morphine on persistent dyspnoea was becoming less certain than previously assumed. This led to our decision to design a three-way crossover study, including not only fentanyl and morphine, but also a placebo arm. We enrolled 58 participants in our study, of which 37 completed all three treatment periods. For both fentanyl compared to placebo and morphine compared to placebo, we found no significant differences with respect to mean and worst daily dyspnoea, sleep quality, health status, mastery, anxiety, hypercapnia or side-effects (expect for a difference in vomiting).

We specifically investigated persistent dyspnoea in stable COPD. The outcomes of our study are in line with other recent RCTs investigating sustained-release morphine and oxycodone during daily life for persistent dyspnoea in COPD and other chronic respiratory diseases [16–20]. On the other hand, there have also been placebo-controlled trials demonstrating a reduction in dyspnoea with opioids [21, 22]. There are several potential factors to take into consideration when trying to explain the mixed results of studies investigating opioids for persistent dyspnoea in COPD.

First, the research setting varies between studies. A recent meta-analysis by Smallwood *et al.* [21] made a distinction between placebo-controlled trials investigating the use of opioids during daily life and those investigating laboratory-based exercise testing. Interestingly, there was a statistically significant and clinically relevant improvement for opioids used for exertional dyspnoea in the laboratory exercise setting, but no significant improvement of dyspnoea in the daily-life setting. This suggests that while opioids can improve (exercise-induced) dyspnoea in chronic respiratory disease under controlled conditions, this effect does not necessarily translate into real-world improvement.

Another meta-analysis, by Liu *et al.* [23], suggested that there may also be a difference between fast-acting and sustained-release opioids for dyspnoea in COPD. The authors found a statistically significant, positive effect on dyspnoea in 13 crossover, placebo-controlled RCTs that investigated short-acting opioids in the majority of participants (72% (136/188) of participants), but they found no positive effect in four parallel placebo-controlled RCTs (706 participants), all investigating sustained-release opioids. However, there has been criticism on the methodology of this meta-analysis, since making a distinction between parallel and crossover trials is not common practice in the performance of a meta-analysis [24].

Another factor to take into account is treatment duration. In our study, participants were treated for 11 days during each treatment period with low-dose fentanyl (12 μg·h^−1^) and sustained-release morphine (10 mg twice daily). Two recent placebo-controlled RCTs, the MORDYC trial and the MABEL trial, investigated morphine over a longer time frame, *i.e.* 4 weeks [19, 20]. In both studies there was no significant change in dyspnoea for the complete study group. However, in the MORDYC trial of Verberkt *et al.* [19], a modest, statistically significant benefit was found of treatment with sustained-release morphine on worst dyspnoea in a subgroup of patients with an mMRC score of 3–4 after 4 weeks. We believe it should be noted that from a pharmacological point of view it is challenging to explain why sustained-release morphine would only be effective after 4 weeks of use, since 11 days of treatment should be enough for the medication to take effect. In addition, most studies demonstrating a positive effect of opioids had a shorter treatment period than our trial, sometimes even a single dose [21]. We also considered whether opioid dosage could play a role in explaining the mixed results of studies. However, there does not seem to be a consistent pattern of higher opioids dosages for those studies finding a positive effect of opioids on dyspnoea [21].

Maybe differences in study populations between studies could also in part explain the differences in studies investigating opioids for dyspnoea. In our study, we included patients with persistent dyspnoea despite receiving optimal care for their underlying COPD. This was assessed by the respiratory physician. We believe it is possible that there are differences in the care patients already received while taking part in studies investigating opioids for dyspnoea in COPD or other chronic respiratory diseases. However, it is challenging to evaluate this systematically. Furthermore, there can also be within-group differences, *i.e.* responders *versus* non-responders. Research has suggested that there may be genetic, psychological and neural factors influencing the chance to be a responder to opioids for dyspnoea [25, 26]. With this in mind, we performed a *post hoc* analysis to see whether we could identify responders to morphine or fentanyl. However, since the number of responders was highest during the placebo treatment period, we did not perform additional analyses on possible factors influencing the chance to be a responder to opioids.

Lastly, we evaluated our outcome measurements. First, health status was included in our study as a secondary outcome. We believe the fact that there was no change in health status is an important signal, since treating persistent dyspnoea is a form of palliative care aimed to improve patients’ health status or quality of life. The crossover design has specific strength to assess health status. For the measurement of dyspnoea, we chose NRS “worst dyspnoea” in addition to NRS “mean dyspnoea” to account for episodic increases in dyspnoea. The NRS scores were reported in a patient diary once daily at a set time. Therefore, participants reported the NRS score “memory based”, and not “moment based” (*i.e.* reporting an NRS during or shortly after a dyspnoea episode). There has been research indicating that patients potentially give a different score for NRS dyspnoea when they score from memory instead of directly in the moment [27]. What we did not measure in our trial was physical activity. Therefore, it could theoretically be possible that participants who experienced improvement of dyspnoea increased their physical activity, leading to the same amount of dyspnoea. In fact, in the recent MABEL trial there was an adjusted mean difference of 9.51 (95% CI 0.54–18.48) min per day of moderate to vigorous physical activity in favour of patients treated with morphine compared to placebo [20]. However, this difference was not statistically significant.

When evaluating all the aforementioned factors possibly explaining the mixed results for opioids for dyspnoea, we propose that the distinction between daily-life settings and laboratory settings is likely to be the most important one. Dyspnoea is a complex symptom, and maybe a unidimensional treatment is unlikely to succeed. This assumption is supported by the fact that trials investigating other medications such as mirtazapine, sertraline and benzodiazepines have found no beneficial effect for breathlessness in advanced cancer and COPD [28–30]. Fortunately, treatment options comprise more than medication, and in recent years there have been publications about non-pharmacological interventions demonstrating positive results. For example, Burge *et al.* [31] performed a systematic review on breathing techniques in patients with serious respiratory disease and found a positive effect on breathlessness and quality of life. In addition, there is some moderate evidence for the use of a handheld fan [32]. Furthermore, holistic, multimodal breathlessness services have been found effective in patients with chronic disease and persistent dyspnoea [33]. A recent feasibility study investigating a similar breathlessness service specifically for patients with COPD found an improvement in mastery and dyspnoea [34].

As indicated, our reasons for investigating fentanyl were two-fold: a potential lower incidence of gastrointestinal side-effects and a possible patient preference compared to oral morphine. Even though we did not find a positive effect of fentanyl on dyspnoea, we were still interested to evaluate side-effects and patient preference. We did find a potential difference in vomiting: this adverse effect occurred during the morphine treatment period (n=5 (11%)), but not during treatment with placebo or fentanyl. Besides vomiting, we found no other difference in adverse events. Interestingly, three of the seven participants who discontinued the trial because of side-effects did so during the placebo treatment. We also found no difference in patient preference; the treatment periods with morphine and fentanyl were preferred by an equal number of participants (both n=13 (38%)). It should be noted that due to the double-dummy design, patients were using a patch and capsules during each treatment period. Therefore, the study design was not fit to investigate preference for a patch over oral medication. However, fentanyl was not selected more often than morphine by participants who continued opioids after finishing with the study medication.

The inclusion of participants in our study took much longer than anticipated. An important reason for this was the COVID-19 pandemic which started shortly after we started study inclusion. In addition, a substantial number of eligible individuals declined participation. The most frequently cited reasons were concerns regarding opioid use, the legal restriction on driving while using opioids under Dutch law and the demanding nature of the study, which required four hospital visits over a 6-week period. To address common concerns with respect to the use of opioids we developed a brief animated video on this subject in collaboration with a Dutch company that specialises in the development of visual patient information (Indiveo; https://indiveo.nl). We asked study participants to provide feedback on this video, and in general it was very well received and appreciated (average score 8 out of 10), suggesting this is a valuable way to inform patients.

An important strength of our study is the crossover design, which is well suited to evaluate patient-reported outcomes. Additionally, the use of a three-arm, double-dummy approach enabled us to contribute to the existing body of evidence on oral morphine for persistent dyspnoea. Simultaneously, this study provided novel data on an alternative opioid and route of administration which, to the best of our knowledge, has not previously been investigated in an RCT for this indication. Finally, this design facilitated the within-subject comparison of treatment preferences, offering further insight into individual patient experiences and acceptability of each intervention.

Our study has some limitations. First, the dropout rate was higher than anticipated (and recruitment more time consuming due to the COVID-19 pandemic), therefore our study was slightly underpowered. However, since we found virtually no differences between the groups, it is unlikely that the inclusion of seven additional participants would have changed the results in any major way. Second, there were instances of missing data, particularly among participants who discontinued the study due to adverse effects, which may have introduced some degree of bias in the intention-to-treat analysis. Third, the study was not powered to allow for a direct comparison between morphine and fentanyl. Therefore, we did not make the statistical comparisons. Lastly, we believe it important to emphasise that we investigated persistent dyspnoea in stable COPD despite optimal pharmacological and non-pharmacological care with an expected life duration of >3 months. Therefore, our results cannot be translated to the end-of-life setting or acute dyspnoea during an exacerbation.

In conclusion, our study found no significant differences in mean and worst daily dyspnoea, sleep quality, health status, mastery, anxiety, hypercapnia or side-effects for transdermal fentanyl nor sustained-release morphine compared to placebo in patients with stable COPD and persistent dyspnoea in a daily-life setting. These findings contribute to a growing body of evidence questioning the use of long-acting opioids for persistent dyspnoea in real life. We advocate a focus on a multimodal approach including non-pharmacological interventions as a more promising avenue to investigate for alleviating dyspnoea in this patient population.

## Data Availability

M. van Dijk (m.van.dijk05@umcg.nl) or H.A.M. Kerstjens (h.a.m.kerstjens@umcg.nl) can be contacted to apply for permission to obtain access to the raw data, data dictionary and statistical code.

## Supplementary material

**Please note:** supplementary material is not edited by the Editorial Office, and is uploaded as it has been supplied by the author. Supplementary material ERJ-01431-2025.SUPPLEMENT

## References

[1] Mahler DA, Selecky PA, Harrod CG, et al. American College of Chest Physicians consensus statement on the management of dyspnea in patients with advanced lung or heart disease. Chest 2010; 137: 674–691. doi:10.1378/chest.09-1543 20202949

[2] Janssen DJ, Wouters EF, Spruit MA. Psychosocial consequences of living with breathlessness due to advanced disease. Curr Opin Support Palliat Care 2015; 9: 232–237. doi:10.1097/SPC.0000000000000146 26125305

[3] van Dijk M, Gan CT, Koster TD, et al. Treatment of severe stable COPD: the multidimensional approach of treatable traits. ERJ Open Res 2020; 6: 00322-2019. doi:10.1183/23120541.00322-2019 32984420 PMC7502698

[4] Abernethy AP, Currow DC, Frith P, et al. Randomised, double blind, placebo controlled crossover trial of sustained release morphine for the management of refractory dyspnoea. BMJ 2003; 327: 523–528. doi:10.1136/bmj.327.7414.523 12958109 PMC192892

[5] Johnson MA, Woodcock AA, Geddes DM. Dihydrocodeine for breathlessness in “pink puffers”. Br Med J (Clin Res Ed) 1983; 286: 675–677. doi:10.1136/bmj.286.6366.675 PMC1547087 6402199

[6] Poole PJ, Veale AG, Black PN. The effect of sustained-release morphine on breathlessness and quality of life in severe chronic obstructive pulmonary disease. Am J Respir Crit Care Med 1998; 157: 1877–1880. doi:10.1164/ajrccm.157.6.9711061 9620921

[7] Richtlijnendatabase. Palliatieve zorg bij COPD. [Palliative care for COPD.] 2021. https://richtlijnendatabase.nl/richtlijn/palliatieve_zorg_bij_copd/startpagina_-_palliatieve_zorg_bij_copd.html Date last accessed: 27 May 2025.

[8] Payne R, Mathias SD, Pasta DJ, et al. Quality of life and cancer pain: satisfaction and side effects with transdermal fentanyl *versus* oral morphine. J Clin Oncol 1998; 16: 1588–1593. doi:10.1200/JCO.1998.16.4.1588 9552070

[9] van Dijk M, Mooren KJM, van den Berg JK, et al. Opioids in patients with COPD and refractory dyspnea: literature review and design of a multicenter double blind study of low dosed morphine and fentanyl (MoreFoRCOPD). BMC Pulm Med 2021; 21: 289. doi:10.1186/s12890-021-01647-8 34507574 PMC8431258

[10] Gift AG, Narsavage G. Validity of the numeric rating scale as a measure of dyspnea. Am J Crit Care 1998; 7: 200–204. doi:10.4037/ajcc1998.7.3.200 9579246

[11] van der Molen T, Willemse BW, Schokker S, et al. Development, validity and responsiveness of the Clinical COPD Questionnaire. Health Qual Life Outcomes 2003; 1: 13. doi:10.1186/1477-7525-1-13 12773199 PMC156640

[12] Wijkstra PJ, TenVergert EM, Van Altena R, et al. Reliability and validity of the Chronic Respiratory Questionnaire (CRQ). Thorax 1994; 49: 465–467. doi:10.1136/thx.49.5.465 8016767 PMC474867

[13] Zigmond AS, Snaith RP. The Hospital Anxiety and Depression Scale. Acta Psychiatr Scand 1983; 67: 361–370. doi:10.1111/j.1600-0447.1983.tb09716.x 6880820

[14] Miller MR, Hankinson J, Brusasco V, et al. Standardisation of spirometry. Eur Respir J 2005; 26: 319–338. doi:10.1183/09031936.05.00034805 16055882

[15] Johnson MJ, Bland JM, Oxberry SG, et al. Clinically important differences in the intensity of chronic refractory breathlessness. J Pain Symptom Manage 2013; 46: 957–963. doi:10.1016/j.jpainsymman.2013.01.011 23608121

[16] Currow D, Louw S, McCloud P, et al. Regular, sustained-release morphine for chronic breathlessness: a multicentre, double-blind, randomised, placebo-controlled trial. Thorax 2020; 75: 50–56. doi:10.1136/thoraxjnl-2019-213681 31558624

[17] Ekstrom M, Ferreira D, Chang S, et al. Effect of regular, low-dose, extended-release morphine on chronic breathlessness in chronic obstructive pulmonary disease: the BEAMS randomized clinical trial. JAMA 2022; 328: 2022–2032. doi:10.1001/jama.2022.20206 36413230 PMC9682426

[18] Ferreira DH, Louw S, McCloud P, et al. Controlled-release oxycodone *vs.* placebo in the treatment of chronic breathlessness – a multisite randomized placebo controlled trial. J Pain Symptom Manage 2020; 59: 581–589. doi:10.1016/j.jpainsymman.2019.10.017 31655189

[19] Verberkt CA, van den Beuken-van Everdingen MHJ, Schols J, et al. Effect of sustained-release morphine for refractory breathlessness in chronic obstructive pulmonary disease on health status: a randomized clinical trial. JAMA Intern Med 2020; 180: 1306–1314. doi:10.1001/jamainternmed.2020.3134 32804188 PMC7432282

[20] Johnson MJ, Williams B, Keerie C, et al. Morphine for chronic breathlessness (MABEL) in the UK: a multi-site, parallel-group, dose titration, double-blind, randomised, placebo-controlled trial. Lancet Respir Med 2025; 13: 967–977. doi:10.1016/S2213-2600(25)00205-X 41033333

[21] Smallwood NE, Pascoe A, Wijsenbeek M, et al. Opioids for the palliation of symptoms in people with serious respiratory illness: a systematic review and meta-analysis. Eur Respir Rev 2024; 33: 230265. doi:10.1183/16000617.0265-2023 39384304 PMC11462312

[22] Ekstrom M, Nilsson F, Abernethy AA, et al. Effects of opioids on breathlessness and exercise capacity in chronic obstructive pulmonary disease. A systematic review. Ann Am Thorac Soc 2015; 12: 1079–1092. doi:10.1513/AnnalsATS.201501-034OC 25803110

[23] Liu M, Xiao W, Du L, et al. Effectiveness and safety of opioids on breathlessness and exercise endurance in patients with chronic obstructive pulmonary disease: a systematic review and meta-analysis of randomised controlled trials. Palliat Med 2023; 37: 1365–1378. doi:10.1177/02692163231194838 37710987

[24] Johnson MJ, Ekstrom M, Janssen DJ, et al. Re: Liu *et al*., Effectiveness and safety of opioids on breathlessness and exercise endurance in patients with chronic obstructive pulmonary disease: a systematic review and meta-analysis of randomised controlled trials. Palliat Med 2024; 38: 400–401. doi:10.1177/02692163241234212 38415671

[25] Currow DC, Quinn S, Ekstrom M, et al. Can variability in the effect of opioids on refractory breathlessness be explained by genetic factors? BMJ Open 2015; 5: e006818. doi:10.1136/bmjopen-2014-006818 PMC4431168 25948405

[26] Abdallah SJ, Faull OK, Wanigasekera V, et al. Opioids for breathlessness: psychological and neural factors influencing response variability. Eur Respir J 2019; 54: 1900275. doi:10.1183/13993003.00275-2019 31073088 PMC6751386

[27] Wade J, Mendonca S, Booth S, et al. Are within-person Numerical Rating Scale (NRS) ratings of breathlessness ‘on average’ valid in advanced disease for patients and for patients’ informal carers? BMJ Open Respir Res 2017; 4: e000235. doi:10.1136/bmjresp-2017-000235 PMC5652535 29071084

[28] Higginson IJ, Brown ST, Oluyase AO, et al. Mirtazapine to alleviate severe breathlessness in patients with COPD or interstitial lung diseases (BETTER-B): an international, multicentre, double-blind, randomised, placebo-controlled, phase 3 mixed-method trial. Lancet Respir Med 2024; 12: 763–774. doi:10.1016/S2213-2600(24)00187-5 39265600

[29] Currow DC, Ekstrom M, Louw S, et al. Sertraline in symptomatic chronic breathlessness: a double blind, randomised trial. Eur Respir J 2019; 53: 1801270. doi:10.1183/13993003.01270-2018 30361250

[30] Simon ST, Higginson IJ, Booth S, et al. Benzodiazepines for the relief of breathlessness in advanced malignant and non-malignant diseases in adults. Cochrane Database Syst Rev 2016; 10: CD007354. doi:10.1002/14651858.CD007354.pub3 27764523 PMC6464146

[31] Burge AT, Gadowski AM, Jones A, et al. Breathing techniques to reduce symptoms in people with serious respiratory illness: a systematic review. Eur Respir Rev 2024; 33: 240012. doi:10.1183/16000617.0012-2024 39477355 PMC11522968

[32] Swan F, Newey A, Bland M, et al. Airflow relieves chronic breathlessness in people with advanced disease: an exploratory systematic review and meta-analyses. Palliat Med 2019; 33: 618–633. doi:10.1177/0269216319835393 30848701

[33] Higginson IJ, Bausewein C, Reilly CC, et al. An integrated palliative and respiratory care service for patients with advanced disease and refractory breathlessness: a randomised controlled trial. Lancet Respir Med 2014; 2: 979–987. doi:10.1016/S2213-2600(14)70226-7 25465642

[34] Mooren K, Wester D, Kerstjens H, et al. Filling the gap: a feasibility study of a COPD-specific breathlessness service. COPD 2022; 19: 324–329. doi:10.1080/15412555.2022.2099821 36004678

