# Supplementary material for: Fentanyl or morphine for persistent dyspnoea in COPD: a multicentre randomised crossover trial
Source: Eur Respir J. 2026 Aug 6;68(2):2501431. doi: 10.1183/13993003.01431-2025 (PMC13446205; doi:10.1183/13993003.01431-2025)
Supplement: Supplementary file 1 [file ERJ-01431-2025.SUPPLEMENT.pdf]

Online supplement:

Table of contents

	Page
**Table S1: In- and exclusion criteria**	**2**
**Table S2: Comorbidities of participants**	**3**
**Table S3: All adverse events registered in the patient diaries**	**4**
**Table S4: Use of supplementary medication**	**5**
**Table S5: Data availability for primary end point analysis**	**6**
Figure S1: NRS questions in the patient diary (in Dutch)	**7**
Figure S2: NRS questions in the patient diary (translated to English)	8

Tabel S1: In- and exclusion criteria.

**Inclusion criteria**
• Age≥40 years
• Read, understood and signed the Informed Consent form
• COPD GOLD class III or IV, according to GOLD criteria: Post-bronchodilatation FEV1/FVC≤70% and FEV1 < 50% of predicted*
• Complaints of refractory dyspnea as established by patient and doctor
• mMRC score≥3
• Life expectancy of≥2 months
• Optimized standard therapy according to Dutch LAN guideline for diagnosis and treatment of COPD
**Exclusion criteria**
• Other severe disease with chronic pain or chronic dyspnea (a non-susbstantial component of left sided heart failure is acceptable)
• Current use of opioids for whatever indication
• Allergy/intolerance for opioids
• Psychiatric disease, not related to severe COPD
• Exacerbation of COPD 8 weeks prior to inclusion or between screening and randomization<br>Problematic (leading to medical help or social problems) substance abuse during the last 5 years<br>Active malignancy, with the exception of planocellular or basal cell carcinoma of the skin
• eGFR <15 ml/min*

COPD = chronic obstructive pulmonary disease; FEV1 = forced expiratory volume in 1 s; FVC = forced vital capacity; GOLD = global initiative for chronic obstructive lung disease; LAN = Lung Alliance The Netherlands; mMRC = modified Medical Research Council Dyspnea Scale; eGFR = estimated Glomerular Filtration Rate. *Measured within 6 months of screening.

Table S2: Comorbidities of participants.

Co-morbidity	Frequency (%)
Cardiovascular disease	
Hypertension	17 (29%)
Coronary artery disease	8 (14%)
Supraventricular tachycardia	8 (14%)
Myocardial infarction	3 (5.2%)
Cerebrovascular accident	3 (5.2%)
Heart failure	2 (3.4%)
Obesity	11 (19%)
Diabetes Mellitus	10 (17%)
OSA/CSA	4 (6.9%)
Asthma	2 (3.4%)
Pulmonary hypertension	2 (3.4%)
Neurological disease	2 (3.4%)
Malignancy	2 (3.4%)
Chronic renal disease	1 (1.7%)
Autoimmune disorder	1 (1.7%)
Psychiatric disease	1 (1.7%)

OSA =Obstructive Sleep Apnea, CSA = Central Sleep Apnea.

Table S3: All adverse events registered in the patient diaries.

Adverse events	Fentanyl (n=48)	Morphine (n=46)	Placebo (n=44)
**Total number of adverse events**	79	83	90
**Number of participants with at least one adverse event**	26 (54%)	22 (48%)	24 (55%)
**Nausea**	11 (22.9%)	15 (32.6%)	12 (27.3%)
**Increased dyspnea**	8 (16.7%)	7 (15.2%)	8 (18.2%)
**Drowsiness**	10 (20.8%)	5 (10.9%)	7 (15.9%)
**Dizziness**	7 (14.6%)	5 (10.9%)	8 (18.2%)
**Obstipation**	6 (12.5%)	9 (19.6%)	4 (9.1%)
**Fatigue**	4 (8.3%)	7 (15.2%)	6 (13.6%)
**Vomiting**	1 (2.1%)	4 (8.7%)	0 (0%)
**Headache**	3 (6.3%)	4 (8.7%)	5 (11.4%)
**Coughing**	1 (2.1%)	1 (2.2%)	5 (11.4%)
**Chills**	2 (4.2%)	2 (4.3%)	3 (6.8%)
**Feeling restless**	2 (4.2%)	1 (2.2%)	4 (9.1%)
**Sleep disturbance**	3 (6.3%)	0 (0%)	4 (9.1%)
**Diarrhea**	1 (2.1%)	1 (2.2%)	4 (9.1%)
**Itching**	3 (6.3%)	2 (4.3%)	1 (2.3%)
**Chest pain**	1 (2.1%)	1 (2.2%)	3 (6.8%)
**Dry mouth**	3 (6.3%)	1 (2.2%)	1 (2.3%)
**Vomiting**	1 (2.1%)	4 (8.7%)	0 (0%)
**Skin irritation from patch**	3 (6.3%)	0 (0%)	1 (2.3%)
**Blurry vision**	1 (2.1%)	2 (4.3%)	1 (2.3%)
**Pain extremities**	0 (0%)	1 (2.2%)	2 (4.5%)
**Sore throat**	2 (4.2%)	1 (2.2%)	0 (0%)
**Stomach ache**	0 (0%)	1 (2.2%)	2 (4.5%)
**Thoracic wall pain**	1 (2.1%)	2 (4.3%)	0 (0%)
**Depression**	0 (0%)	1 (2.2%)	1 (2.3%)
**Edema extremities**	0 (0%)	1 (2.2%)	1 (2.3%)
**Flatulence**	0 (0%)	2 (4.3%)	0 (0%)
**Forgetfullness**	0 (0%)	1 (2.2%)	1 (2.3%)
**GE reflux**	0 (0%)	1 (2.2%)	1 (2.3%)
**Muscle cramps**	0 (0%)	1 (2.2%)	1 (2.3%)
**Rash**	0 (0%)	0 (0%)	1 (2.3%)
**Restless legs**	0 (0%)	1 (2.2%)	1 (2.3%)
**Altered taste**	1 (2.1%)	0 (0%)	0 (0%)
**Altered smell**	0 (0%)	1 (2.2%)	0 (0%)
**Common cold**	0 (0%)	0 (0%)	1 (2.3%)
**Feeling warm**	1 (2.1%)	0 (0%)	0 (0%)
**Loss of appetite**	1 (2.1%)	0 (0%)	0 (0%)
**Muscle ache**	0 (0%)	0 (0%)	1 (2.3%)
**Night sweats**	1 (2.1%)	0 (0%)	0 (0%)
**Tachycardia**	1 (2.1%)	0 (0%)	0 (0%)
**Tingling sensation hands**	0 (0%)	1 (2.2%)	0 (0%)
**Vivid dreams**	0 (0%)	1 (2.2%)	0 (0%)

Table S4: Use of supplementary medication

Use of supplementary medication	Fentanyl (n=48)	Morphine (n=44)	Placebo (n=44)
**Macrogol/electrolytes**			
**No. of participants (%)**	22 (46)	22 (50%)	17 (39%)
**No. of sachets**	10 [1-14]	11 [1-20]	6 [1-14]
**Metoclopramide**			
**No. of participants (%)**	8 (17%)	8 (18%)	8 (18%)
**No. of capsules**	2 [1-11]	5 [1-13]	1 [1-8]

Number of sachets and capsules described as median [min-max].

Table S5: Data availability for primary end point analysis

Inclusion: 58 participants, 59 randomizations

Statistical analysis of fentanyl vs placebo could be performed for n=39.
Statistical analysis of morphine vs placebo could be performed for n=38.

**Fentanyl - Excluded from analysis primary endpoint**	**(n = 20)**
Did not start, opioid use missed during screening	(n= 1)
Exacerbation during treatment period	(n= 5)
Data incomplete	(n= 4)
No comparison with placebo possible	(n= 4)
Study discontinued before treatment with Fentanyl	(n= 6)
**Morphine - Excluded from analysis primary endpoint**	**(n = 21)**
Exacerbation during treatment period	(n= 5)
Data incomplete	(n= 1)
No comparison with placebo possible	(n= 4)
Study discontinued before treatment with morphine	(n= 11)
**Placebo – No data available for analysis**	**(n = 17)**
Did not start, second thoughts about side effects	(n= 1)
Exacerbation during treatment period	(n= 3)
Data incomplete	(n= 3)
Study discontinued before treatment with Placebo	(n= 10)

Figure S1: NRS questions in the patient diary (in Dutch)

**Wilt u met een cijfer aangeven hoe erg u *gemiddeld* last heeft gehad van kortademigheid in de afgelopen 24 uur?**
0 1 2 3 4 5 6 7 8 9 10<br>0 =helemaal niet kortademig. 10= ergst mogelijke kortademigheid denkbaar.
**Wilt u met een cijfer aangeven hoe erg de *ergste mate* van kortademigheid was die u de laatste 24 uur gevoeld heeft?**
0 1 2 3 4 5 6 7 8 9 10<br>0 =helemaal niet kortademig. 10= ergst mogelijke kortademigheid denkbaar.
**Wilt u met een cijfer aangeven hoe u geslapen heeft de afgelopen nacht?**
0 1 2 3 4 5 6 7 8 9 10<br>0 = de beste slaap denkbaar. 10 = slechtste slaap ooit.
**Heeft u last gehad van bijwerkingen?<br>Zo ja, welke klachten had u?**

Figure S2: NRS questions in the patient diary (translated to English)

**Please indicate with a number how much, on average, you have suffered from dyspnea in the past 24 hours**
0 1 2 3 4 5 6 7 8 9 10<br><br>0 = No dyspnea at all 10= The worst dyspnea you can imagine
**Please indicate with a number how severe the worst level of dyspnea you have felt in the past 24 hours was**
0 1 2 3 4 5 6 7 8 9 10<br><br>0 = No dyspnea at all 10= The worst dyspnea you can imagine
**Could you rate how well you have slept last night?**
0 1 2 3 4 5 6 7 8 9 10<br><br>0 = the best night of sleep you can imagine 10 = the worst night of sleep you can imagine
**Did you experience any side effects?**<br><br>**If so, what complaints did you experience?**
